# Central Nervous System Miliary Brain Metastasis Secondary to Breast Cancer

**DOI:** 10.7759/cureus.9649

**Published:** 2020-08-10

**Authors:** Gabriel Santos Vázquez, Ricardo Garcia Cázarez, Erick Moreno Pizarro, Aarón Emanuel Serrano Padilla, Juan Carlos Plascencia Salcedo

**Affiliations:** 1 Medicine, University of Guanajuato, León, MEX; 2 Endovascular Neurologist, Instituto de Seguridad y Servicios Sociales de los Trabajadores del Estado (ISSSTE), León, MEX; 3 Academic Unit of Medicine, Autonomous University of Nayarit, Nayarit, MEX

**Keywords:** cerebral milliary metastasis, cerebral calcification, breast cancer, cognitive impairment, brain metastasis, miliary metastasis, literature review

## Abstract

Miliary metastasis to the central nervous system (CNS) is a rare presentation of metastasis mainly found in primary adenocarcinoma of the lung. Its association with breast cancer is even less frequent. We present the case of a 50-year-old female patient diagnosed in 2010 with stage IIA infiltrating ductal breast cancer RE (-), RP (+), HER 2 (-), HER2 NEU (+). She was treated with modified radical left breast mastectomy, radiation therapy, and chemotherapy. Her condition began presenting oppressive frontal headache without irradiation, predominantly in the evening, intensity 8/10, which decreased when sleeping and was exacerbated with stressful situations, in addition to progressive cognitive deterioration. Simple and contrasted computed tomography (CT) of the skull and thoracoabdominal were requested, showing multiple micronodular lesions with calcium density in the brain parenchyma, left pleural effusion, hypo and hyperdense lesions in the liver parenchyma, as well as osteoblastic lesions in the lumbar spine. Simple and contrasted magnetic resonance imaging (MRI) of the skull showed multiple supra and infratentorial intra-axial lesions. The most frequent associated symptom with miliary metastasis is cognitive impairment. Miliary metastasis, confirmed by imaging studies and histopathology, requires the ruling out of other causes of this calcification pattern, such as neurocysticercosis, due to specific treatment for each pathology.

## Introduction and background

Miliary metastasis was first described in 1951 as "carcinomatous encephalitis." It was described as multiple plaques formed from perivascular distribution [1]. It is a rare presentation in breast cancer. At this time, its mechanism of occurrence is not fully known [2-3]. Physiological (pineal gland, choroid plexus, habenula, falx cerebri, tentorium, among others), and pathological (tuberculosis, cysticercosis, TORCH (Toxoplasma gondii, others (including Treponema pallidum, Listeria, Varicella, and parvovirus B19), rubella virus, cytomegalovirus (CMV), and herpes simplex virus (HSV)) disease, chronic viral encephalitis, Fahr's disease, thyroid or parathyroid disease) [4] calcifications could complicate the clinical diagnosis of miliary metastasis since they present with similar symptoms such as hemiparesis, dysarthria, short/long-term memory loss, seizures, language abnormalities, ataxia, dementia, psychosis, or headache [2,5]. The objective of this work is to describe a case of central nervous system miliary metastasis secondary to breast cancer, as well as a literature review on this topic.

Patients and methodology

A literature review of both English and Spanish was performed in the Medline databases using the following keywords: "miliary calcifications brain", "miliary brain metastases", "metastasic breast cancer", and "miliary brain calcifications", as well as their respective keywords in Spanish. Independent of the primary tumor strain, all case reports since 1988 to 2019 on miliary brain metastases in the central nervous system, were selected and their clinical characteristics described. Likewise, a patient's case was reported and sent to the neurology service of our unit, where this diagnosis was made.

## Review

Case presentation

A 50-year-old female nurse, with genetic load for diabetes mellitus, denied smoking and drug use, occasional alcoholism, and allergic to sulfonamides. She denied chronic-degenerative diseases, transfusions, or trauma-related conditions. She was diagnosed with breast cancer in 2010, treated with a modified radical mastectomy of the left breast. Histological variety described as an invasive ductal carcinoma stage IIA RE (-), RP (+), HER 2 (-), HER2 NEU (+). She was administered adjuvant chemotherapy with anthracyclines and taxanes sequentially, eight cycles, 25 sessions of radiotherapy, tamoxifen for five years, with extended adjuvant anastrozole and exemestane to date.

Symptoms began on July 30, 2019, as she described an acute frontal headache without irradiation, predominantly in the evening, intensity 8/10. It decreased while sleeping and exacerbated with stressful situations, without other accompanying symptoms. On August 5, 2019, she attended her checkup at the oncology medical visit, commenting on her symptoms. In addition, the physical examination noted disorientation in time and space and a language disorder being inconsistent and inappropriate. It was decided to hospitalize her for the complete study protocol. Regarding the initial exploration, her vital signs were within normal parameters, normocephalus, without obvious lesions, rhythmic precordium without murmurs, left basal hypoventilation without crackles, and decreased vocal resonance. Abdomen and limbs were without abnormalities.

Upon neurological examination, the patient showed alterations in mental state, with disorientation in time and space. Folstein’s mini-mental test was 19/30, with impaired memory, abstraction, judgment, and language. Cranial nerve examination showed no abnormalities. There was motor function with preserved tone and trophism in all extremities, strength was 4/5 proximal and distal in the right arm, 5/5 in all the others. Deep tendón reflexes were ++/++++ and the Babinski sign was negative. The sensory system was not objectively evaluated due to the mental state of the patient. The cerebellar function showed bilateral eumetry and eudiadocinesia, without pathological nystagmus, as well as normal gait. There were no meningeal irritation signs. Pathologic reflexes were present: sucking, palmomental, and grasping.

**Figure 1:**
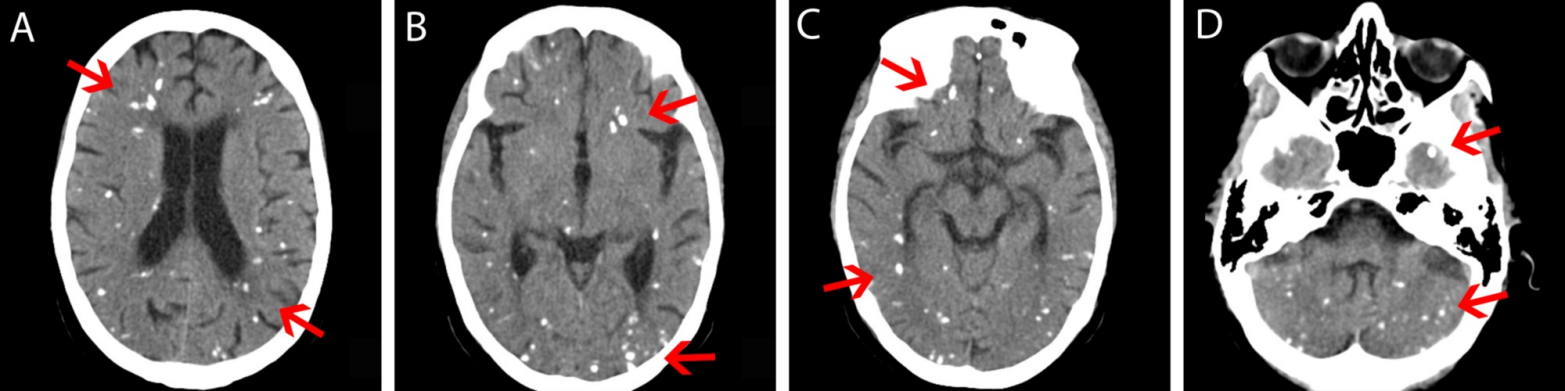
Transverse cerebral CT scan Red arrows in A-D: Micronodular lesions with calcium density distributed throughout the brain parenchyma. With gray and white substance affection. CT: computed tomography

The patient was admitted with a diagnosis of an acute confusional state. A simple and contrasted CT scan of the skull was performed, showing multiple micronodular lesions in the entire cerebral parenchyma, both white and gray matter, without meningeal enhancement. (Figure 1, Appendix 1). Complementary tests showed hematic cytometry, blood chemistry of three elements, serum electrolytes, and thyroid function test within normal parameters. Liver function tests reported total bilirubin 0.3 mg/dL, conjugated bilirubin 0.3 mg/dL, aspartate aminotransferase (AST) 43 U/L, alanine transaminase (ALT) 52 U/L, gamma-glutamyl transferase (GGT) 158 U/L, alkaline phosphatase 100 U/L, albumin 3 g/dL, parathormone levels 22.7 pgr/mL (4-58.1). The cerebrospinal fluid analysis was reported as acellular, the acid-fast Bacillus (AFB) test was negative, cysticercus antigen was negative, proteins 50 mg/dL. The serology was as follows: CMV immunoglobulin G (IgG) >180, CMV IgM <5, toxoplasmosis IgG 22.5, toxoplasmosis IgM <3, rubella IgG 13.8, and rubella IgM 11.7. Anti-HIV 1 and 2 (anti-human immunodeficiency virus 1 and 2) were negative, hepatitis C was negative, and hepatitis B surface antigen was negative. With these results and due to the clinical presentation dexamethasone was started.

**Figure 2:**
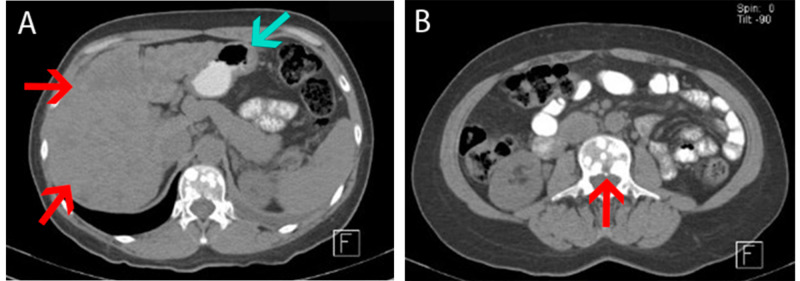
Abdominal CT scan with corresponding images of metastatic lesions A: Multiple hypo- and hyperdense lesions distributed in the liver parenchyma (red arrows), as well as left basal pleural effusion (blue arrow); B: Hyperdense lesion at the L1 level (red arrow) CT: computed tomography

**Figure 3:**
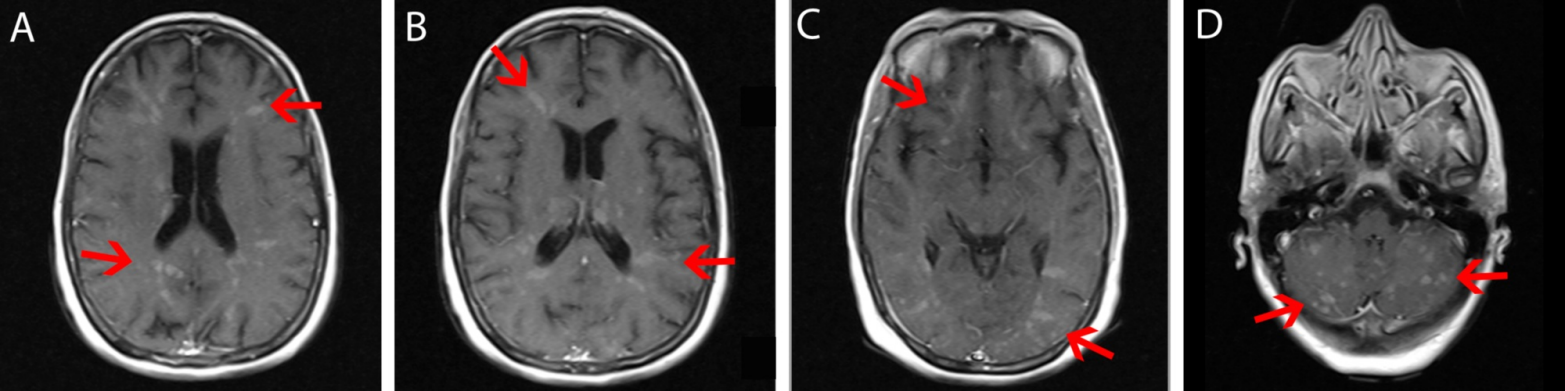
Cerebral MRI (T1) A-D: Multiple hyperintense micronodular lesions in T1 distributed heterogeneously in the brain parenchyma (red arrows). The white matter lesions showed enhancement after administration of gadolinium. MRI: magnetic resonance imaging

Simple and contrasted thoracoabdominal CT was requested, which showed left pleural effusion, multiple hypodense hepatic lesions with contrast, as well as osteoblastic lesions at the lumbar spine level (Figure 2). MRI of the skull showed multiple intra-axial supra and infratentorial lesions with gadolinium enhancement in the T1 sequence (Figure 3).

The patient was treated with prophylactic anticonvulsants and antiedema steroids and was transferred to a tertiary center to receive holocranial radiotherapy.

Discussion

Breast cancer is the most common cancer in women in the United States. In 2018, the World Health Organization (WHO) estimated that it affects 2.1 million women each year, 627,000 women died of breast cancer, in addition to it accounting for 15% of all cancer deaths among women. Nearly 30% of new breast cancer diagnoses have already spread to regional lymph nodes and 5% of these occur with metastases at the time of presentation, with the median age of diagnosis being at 50 years [5-6]. Breast cancer can be classified by microarray techniques into several intrinsic subtypes: luminal A, luminal B, enriched with HER2, and triple-negative [7]. Central nervous system (CNS) metastasis is most frequently observed in the following subtypes of breast cancer: Triple-negative, shows a higher incidence of visceral and cerebral tumor metastasis (46%), HER2-positive (5-30%), as well as TP-53 positive; the last one of these was reported with a 38% higher probability of occurrence as compared to TP-53 negative [5-6]. Metastatic lesions have been related to the initial site of appearance; it is more common after initial metastasis to the bone (26.7%). Once pleural metastasis has been established, the disseminated disease is more common, including CNS compromise in 63.6% [8].

There have been 26 cases of miliary metastasis to the CNS reported to date. The most frequent primary tumor was lung (61.54%), followed by breast and unknown origin, with 11.54% each.

The clinical presentation of miliary metastasis to the CNS is heterogeneous, the most frequent symptoms, in general, were cognitive impairment in 28%. In patients with primary breast tumors, the most frequent symptoms were psychiatric alterations and language disorders.

To date, the most associated cancer with military metastasis to the central nervous system is lung cancer. The importance of our case report lies in the rarity of its association and the clinical description of its manifestations. From all the reported cases, this is the third worldwide with a primary origin in the breast. Besides, the most frequent symptomatology has been described according to each lineage to date.

Among the differential diagnoses to be considered in our population are neurocysticercosis and CNS tuberculosis, however, clinically, the first one usually presents with epilepsy, while tuberculosis tends to manifest more frequently as basal arachnoiditis, affecting the lower cranial nerves [9-10]. In consideration of these findings, data from CT, MRI, and cerebrospinal fluid (CSF) study, we excluded these diagnostic possibilities.

Table 1 lists the 26 cases of miliary metastasis to the CNS while Table 2 lists the frequency of the primary origin of miliary metastasis to the CNS.

**Table 1:** Cases reported in the literature to date of miliary metastasis to the central nervous system F: female; M: male

Reference and year	Gender and age	Primary tumor site	Histological variant	Symptoms
Madow and Alpers (1951)[1]	55/M	Lung	Adenocarcinoma	Psychiatric Hemiparesis Aphasia Convulsions
	48/M	Unknown	Adenocarcinoma	Psychiatric Convulsions
	47/M	Lung	Adenocarcinoma	Psychiatric Hemiparesis Convulsions
	31/F	Lung	Adenocarcinoma	Headache Convulsions Psychiatric
Fukuda et al (1988)[11]	60/F	Lung	Adenocarcinoma	Hemiparesis Cognitive impairment
Ara Callizo et al (1989) [12]	61/M	Pancreas	Acinar Cell Carcinoma	Confusion Hemiparesis
Nemzek et al (1993)[13]	59/F	Lung	Small cell carcinoma	Bradypsychia Convulsions
Yamazaki et al (1993)[3]	58/M	Lung	Adenocarcinoma	Cognitive Impairment Hemiparesis
Shirai et al (1997)[14]	68/M	Neck (salivary gland)	Adenocarcinoma	Headache Hemiparesis
Bushan et al (1997)[15]	69/M	Unknown	Small Cell Carcinoma	Vertigo Cognitive Impairment
Nakamura et al (2001)[16]	44/M	Lung	Adenocarcinoma	Psychiatric
Miyamura et al (2002) [17]	75/M	Lung	Adenocarcinoma	Cognitive Impairment Convulsions
Rivas et al (2005) [18]	79/F	Unknown	Adenocarcinoma	Visual Hallucinations Extrapyramidal Symptoms
Ota et al (2006) [19]	58/M	Lung	Adenocarcinoma	Psychiatric
Ogawa et al (2007)[17]	82/F	Lung	Adenocarcinoma	Cognitive Impairment
Iguchi et al (2007)[20]	66/M	Lung	Adenocarcinoma	Cognitive Impairment
Inomata et al (2012)[21]	68/F	Lung	Adenocarcinoma	Headache Ataxia
Kahveci et al (2012) [22]	52/M	Lung	Adenocarcinoma	Poor Cooperation
Bekiesinska et al (2013)[23]	52/M	Lung	Adenocarcinoma	Cognitive Impairment
Dumoulin et al (2015)[24]	58/M	Lung	Adenocarcinoma	Cognitive Impairment
Zhang et al (2016)[25]	3/F	Cerebellopontine Angle	Teratoid rhabdoid tumor	Facial Paralysis Hearing Impairment
	27/M	Thalamus	Glioma	Facial Paralysis Hearing Impairment
de Ceuster et al (2016) [26]	38/F	Breast	Unspecified	Dysarthria Ataxia
Cools et al (2019)[2]	48/F	Breast	Unspecified	Hallucinations Delirium Aphasia
Kurihara et al (2019) [27]	74/F	Lung	Adenocarcinoma	Cognitive Impairment Akinetic Mutism
The case reported in this review (2020)	50/F	Breast	Invasive Ductal	Headache Cognitive Impairment

**Table 2:** Frequency of primary origin of miliary metastasis to the central nervous system

Primary site	Frequency % (n)
Lung	61.54 (16)
Breast	11.54 (3)
Unknown	11.54 (3)
Thalamus-pontine	7.69 (2)
Pancreas	3.85 (1)
Neck	3.85 (1)
Total	100 (26)

The main weakness of our study was the lack of a confirmatory histopathological study. However, the most relevant differential diagnoses were excluded.

## Conclusions

CNS metastases are common among cancers. Miliary metastasis is rare, with breast cancer being even less common. Therefore, its finding requires differential diagnoses with other entities, which radically changes the treatment and prognosis of these patients.

## Figures and Tables

**Figure 4:**
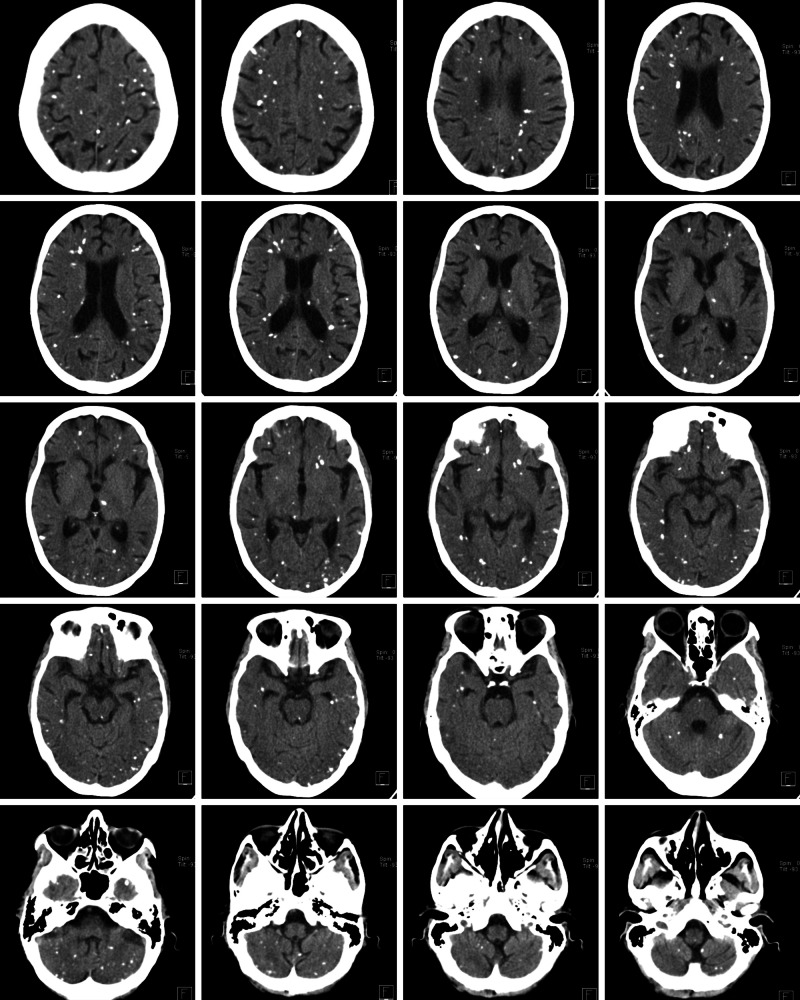
Cerebral transversal CT scan (20 slices) Micronodular lesions throughout all the brain parenchyma CT: computed tomography

## Appendices

Appendix 1 
